# Validating a Minimally Invasive Tissue Sampling (MITS) Method in Determining Cause of Death in Stillbirths and Neonates

**DOI:** 10.3390/children8121095

**Published:** 2021-11-27

**Authors:** Naanlep Matthew Tanko, Ibrayimov Bakytkaly, Alpamys Issanov, Dimitri Poddighe, Milan Terzic

**Affiliations:** 1 Department of Biomedical Sciences, School of Medicine, Nazarbayev University, Nur Sultan 010000, Kazakhstan; alpamys.issanov@nu.edu.kz; 2 Clinical Academic Department of Laboratory Medicine, Pathology and Genetics, University Medical Center, Nur Sultan 010000, Kazakhstan; bakytkali.Ibrayimov@umc.org.kz; 3 Department of Medicine, School of Medicine, Nazarbayev University, Nur Sultan 010000, Kazakhstan; dimitri.poddighe@nu.edu.kz (D.P.); milan.terzic@nu.edu.kz (M.T.); 4 Clinical Academic Department of Pediatrics, University Medical Center, Nur Sultan 010000, Kazakhstan; 5 Clinical Academic Department of Women’s Health, University Medical Center, Nur Sultan 010000, Kazakhstan; 6 Department of Obstetrics, Gynecology and Reproductive Sciences, School of Medicine, University of Pittsburgh, Pittsburgh, PA 15213, USA

**Keywords:** MITS, cause of death, stillbirths, neonates

## Abstract

Complete diagnostic autopsy (CDA) remains the gold standard and a valuable technique for determining cause of death. It is a source of health statistics that can be used to measure health care services’ quality, unraveling important information on disease processes, particularly in emerging and unknown diseases. It can also be a vital tool for medical education and biomedical research. However, autopsy rates have been declining globally. There is an urgent need to develop and validate alternative methods in different settings to provide reliable information on cause of death. In this study, we aimed to determine cause of death (KazCoDe) in neonates and infants using minimally invasive tissue sampling (MITS), and to compare these results with those of CDA. We conducted MITS and CDA sequentially on 24 deceased children at the Pathological Bureau of the Akimat of the city of Nur-Sultan. Clinical data of the study subjects were extracted from their clinical records. During both procedures, brain, liver and lung tissues were collected for pathological diagnosis. Fifteen (62.5%) and nine (37.5%) were stillbirths and neonates, respectively. Eight (33.3%) were females and 16 (66.7%) were males. MITS diagnosis of cause of death was concordant with CDA diagnosis in 83.3% out of the 24 cases when considering the immediate and underlying causes of death and reviewing all the clinical and laboratory test results as part of the diagnostic evaluation to arrive at a cause of death (ICD-PM). We concluded that MITS is a valuable and reliable method for cause of death diagnosis in stillbirths and neonates, which can contribute vital mortality statistics in children in the absence of CDA.

## 1. Introduction

Postmortem examination remains a valuable technique in providing mortality statistics that can be used to design and implement health care programs and for monitoring their effectiveness. The data generated can also be used for the advancement of science and medical education. Complete diagnostic autopsy (CDA) has been proven irrefutably as the gold standard for the determination of cause of death in many cases over the years [1]. The importance of CDA has been demonstrated clearly over the last two decades in unraveling the pathogenesis of unknown and emerging diseases caused by coronaviruses. For example, Sessa et al. and Salerno et al. [2,3] recently conducted two detailed reviews. They included all case reports, case series, retrospective and prospective studies, letters to the editors, and reviews that focused on MERS-CoV, SARS-CoV, SARS-CoV-2, and autopsy; CDA provided a substantial amount of information on the pathology of these pathogens that could have been missed without autopsy. In their conclusion, they recommended that we still need autopsies in the 21st century and the dictum “to learn from the dead” should not be an exception but the rule, particularly regarding unknown and emerging diseases. Despite these benefits, autopsy rates have been declining over the years globally. This decline is of great concern to public health, as mortality data in communities of the majority of the world’s population remain largely unrecorded. Worst still is the fact that low- and middle-income countries (LMIC), which bear most of the global burden of disease, suffer more from the lack of these vital statistics [4,5,6].

In developing countries, the World Health Organization’s (WHO) approved criteria for verbal autopsies have been used as an alternative to determine cause of death, but these have shown significant levels of imprecision and a high degree of misclassification errors [7,8]. In Kazakhstan, the observed decline in autopsy rates is no exception, particularly as regards death occurring in adults (unpublished report). The determination of cause of death has largely been based on clinical information. We are of the opinion that the time for validating alternative methods of obtaining tissues for cause of death diagnosis has come. The diagnostic accuracy of the minimally invasive tissue sampling (MITS) procedure has been tested against CDA in some few previous studies in adults, children, stillbirths and neonates [9,10,11]. The great potential for MITS to add reliable cause of death information was also confirmed in one recently published study in a population in Malawi [12]. To date, no large-scale, multicenter studies have been conducted to validate the diagnostic accuracy of MITS when compared to CDA. Nowadays, with the challenge of COVID-19 as a highly transmissible disease and other emerging infectious diseases, complete diagnostic autopsy in these patients may not be feasible due to the potential high risk of infection, and the only way to determine a cause of death remains MITS [13].

To the best of our knowledge, no previous MITS-based studies have been conducted in Kazakhstan. Our population is, therefore, a MITS-naïve population. In this observational study, we aimed to define the reliability of MITS in precise identification of causes of death in stillbirths and neonates. We employed the WHO International Classification of Disease codes for assigning cause of death in stillbirths and neonatal deaths (ICD-PM).

## 2. Materials and Methods

### 2.1. Participants and Population Characteristics

We performed the coupled MITS and CDA procedures at the Pathological Bureau of the Akimat of the city of Nur-Sultan (Astana). Pathological evaluation of samples was carried out at this center. In Kazakhstan, the law mandates an autopsy in all pediatric deaths from 22 gestational weeks to 11 months of age (Article 56 of the Health Code of the Republic of Kazakhstan).

### 2.2. Setting and Recruitment

The study was carried out between November 2020 and June 2021. Enrolment of study subjects was expected to begin in January of 2020, but had to be extended due to the COVID-19 pandemic and its attendant quarantine measures that were imposed for several months in 2020. All subjects that met the eligibility criteria were recruited into the study. The University Medical Center (UMC) Expert Commission on Bioethics granted a waiver of documentation of informed consent for both CDA and MITS, and ethical approval was obtained before study subjects were enrolled (Certificate/Decision; No. 6 of the Ethical Commission of the UMC of 20 September 2019).

### 2.3. Eligibility and Exclusion Criteria

Patients from 22 gestational weeks to 11 months in whom autopsy was requested as part of the medical evaluation for cause of death determination were considered eligible for enrolment into the study. Subjects excluded from the study were those who died of traumatic incidents, fire burns, drowning, severely macerated or autolyzed bodies. We did not exclude any subject based on the place of death, whether it was in the hospital or at home.

### 2.4. Data Collection Method

Patient demographic information was recorded in the MITS data collection form. The MITS assistant recorded the patients’ age, sex and all relevant clinical information in the form.

### 2.5. Autopsy Procedures: MITS and CDA

#### 2.5.1. Personal Protective Equipment (PPE)

For both MITS and CDA, pathologists and assistants were properly donned in PPE, which included single-used fluid resistant, long-sleeved gowns, surgical masks, disposable surgical caps, eye goggles, autopsy gloves and closed shoes with shoe covers. All the procedures were carried out in standard autopsy rooms with proper ventilation and illumination, as recommended by hospital protocols.

#### 2.5.2. Biopsy Needles

The Bard Monopty^TM^ 16G, 160 mm (brain), 16G 100 mm (lung/thorax, liver), 16G 10 mL syringe (blood), 18G spinal puncture needle and 10 mL syringe (CSF) were used for specimen collection according to the MITs protocols.

Trained study staff (MITS specialists and MITS assistants) performed the coupled MITS and CDA procedures. One pair (pathologist and assistant) performed only MITS without prior review of the clinical records and other relevant premortem information and then left the autopsy room for the second pair of trained staff to perform the CDA. The CDA team had the opportunity to review all the clinical data and relevant information, as is usually carried out in routine practice. The MITS procedure was carried out according to the protocol outlined in the MITS standard operating procedures (SOPs) in the procedure handbook and adapted where necessary to hospital protocols.

External body inspection for visible lesions and deformities, skin maceration and its grade were all carried out systematically according to the MITS protocols. Disinfection and sterilization of the puncture sides were performed according to the protocols in the procedure manual. In brief, the MITS specialists cleaned the area to be punctured with abundant alcohol. Cleaning was performed with circular movements from the center to the periphery. The alcohol was allowed to dry for 5 min. The area was then cleaned with abundant iodine solution, repeating the same circular movements from the center to the periphery. The iodine solution was allowed to dry for 5 min before the puncture procedure was performed. Percutaneous needle biopsies of the liver, lungs and brain tissues were conducted using the Bard Monopty^TM^ disposable biopsy needles described above.

Six cores of tissue from each organ (i.e., the liver, the brain and from the left and right lungs) were obtained. The specimens were fixed immediately in 10% buffered formalin for 4–6 h in special jars provided by the MITS Alliance, and then, subjected to routine procedure of tissue processing according to hospital protocols for pathological analysis of tissues. Additional tissue cores were stored in cryogenic vials in RNA*later* at −20 °C in a freezer for molecular analysis, if it was indicated. For the brain, with the body in the supine position, the head was turned laterally and the needle was inserted 3–4 cm below the occipital protuberance at an angle of 30° to the skin of the back and advanced towards the orbital cavities until the needle was freely advancing, indicating that it was in the cranial cavity. The automatic needle was then released to take a core of brain tissue. For the lung tissues, the punctures were performed in the mid axillary line at the upper region of the right or left thorax oriented towards the head. Once the estimated depth was reached (approximately 1.5–2 cm), the specialists released the automatic needle to obtain lung tissue. This was repeated at different angles using the same puncture site to take representative samples from all the lobes of the lungs on the left and the right. In total, 12 cores of lung tissues were obtained. The liver tissue was obtained in a similar approach with the needle placed in the mid axillary line in any of the last 3 intercostal spaces. The needle was oriented at 30° to the cranial position and 15° laterally. Six cores of liver tissue were obtained.

Next, 10 mL of blood was collected from the supraclavicular vein or from the right ventricle of the heart using a 16G needle and 10 mL syringe. Briefly, for the supraclavicular approach, the needle was inserted 1 cm lateral to the mid-clavicular line above or below the clavicle. Occasionally, we encountered difficulty obtaining blood from this approach and we inserted the needed directly into the right ventricle of the heart to obtain blood. At the end of the puncture procedures, Monsel’s solution (hemostatic solution) was used to stop bleeding before the CDA was performed. Where this was not available, sustained pressure using gauze reduced the amount of bleeding.

In addition, 10 mL of cerebrospinal fluid (CSF) was collected via a puncture at the nape of the neck below the occipital protuberance using an 18G needle and 10 mL syringe. In brief, for CSF collection, with the body in the supine position, the head was turned laterally and the needle was inserted through the skin, leaning towards the orbital cavities at an angle of approximately 75° to the skin of the back at the midline and 2–3 cm below the occipital protuberance. Both samples were collected for microbiological analysis if an infectious cause was strongly suspected. In cases with a premortem microbiology and cytogenetic test results, we retrieved such information from their medical records. A third trained staff (MITS assistant) managed the data entry in the MITS specimen collection forms. In addition, placental tissue sampling and processing was performed according to the Amsterdam Criteria in all stillbirths who had placenta available.

The CDA team then performed CDA after the MITS team had completed the needle sampling procedures. In brief, with the bodies in the supine position, external examination, and measurements of lengths, mid arm and head circumferences were all documented. A midline incision was made, and skin and subcutaneous tissues were dissected away and retracted to access the thoracic and abdominal organs. The anterior rib cage was removed to access the mediastinal structures. The organs were removed en bloc (Rokitansky method). The brain was removed through the anterior fontanelle approach. Biopsies were also taken from the same organs and body fluids during the CDA (i.e., liver, brain and lungs, CSF and blood). Both pairs of biopsy materials were submitted to the pathology laboratory for histological studies. We did not perform immunohistochemical studies, as this was not necessary. A cause of death diagnosis was made using microbiology, cytogenetic and morphological studies of tissues and body fluids.

#### 2.5.3. MITS Tissue Adequacy Assessment

For the solid tissues, i.e., brain, lungs and liver, needle biopsies samples were adequate in all 24 cases (100%).

#### 2.5.4. Determination of Cause of Death

The MITS team performed blinded pathological evaluation to assess the diagnostic performance of MITS before a consensus on cause of death was determined for each case by both teams. A definitive cause of death or causal chain of events leading to death was assigned based on the presence and severity of the pathological features, cytogenetic test (when it was available) result as well as reviewing the maternal, fetal or perinatal co-morbid conditions. In assigning the cause of death, the MITS team independently evaluated all the results of histology, microbiology and cytogenetic tests on the samples collected during MITS. The CDA team performed a similar independent evaluation and assigned a cause of death. Both teams then discussed the results together (in a cause of death panel) and studied the agreement or discrepancy in the diagnosis The diagnostic performance of MITS in children with congenital anomalies was limited when only the histopathological features were evaluated. The histopathological features in the brain and liver tissues were mainly non-specific and did not contribute significantly to the pathological diagnosis of the cause of death. The lung tissues, however, showed significant features that contributed to both the immediate and underlying cause of death. Bronchopulmonary dysplasia, pneumonic features and alveolar hyaline membranes were a common finding in the lungs of these still births and neonates. However, the diagnostic performance of MITS and concordance with the CDA diagnosis increased significantly when clinical information and the results of laboratory tests were used as part of the evaluation to identify the immediate and underlying causes of death. The WHO application of ICD-10 to deaths during the perinatal period (ICD-PM) codification was used in all cases.

### 2.6. Statistical Methods

We used descriptive statistics of the patients and presented the results either as means and standard deviations for normally distributed continuous variables, or as medians and interquartile ranges for non-normal continuous variables, while categorical variables were summarized as frequencies and percentages. Concordance rates of MITS to CDA were calculated as the percentage of matching diagnosed cases between MITS and CDA methods. Agreement in ICD-10 code diagnoses between MITS and CAD were assessed using McNemar’s test for matched data and Cohen’s kappa agreement test. Non-significant McNemar’s test statistic and statistically significant Cohen’s kappa agreement estimate indicated concordance of both methods.

## 3. Results

We performed coupled MITS and CDA approaches on 24 stillbirths and neonates (15 stillbirths and 9 neonates). Eight (33.3%) and 16 (66.7%) were females and males, respectively. The mean weight ± SD was 2244 g ± 1420, and the median (IQR) was 2201 (1250–3027). The mean length (cm) ± SD was 46.2 ± 11.9; the median (IQR) was 48.5 (38.3–53). Gestational weeks (mean ± SD) was 31.8 ± 5.5; their median age in days was (IQR) was 20 (13–34). The mean time between death and autopsy procedures (both MITS and CDA) was 29.2 ± 25.3 (SD) hours (Table 1).

Table 2 shows causes of death as determined by complete diagnostic autopsy. Intrauterine hypoxia was responsible for 54.2% of the causes of death among the 24 cases. Congenital malformations, deformations and chromosomal abnormalities accounted for 20.9%. The remaining 24.9% was attributed to primary atelectasis of the newborn, bacterial sepsis and disorders of urea cycle metabolism. In Table 3, the concordance rates between the complete autopsy diagnoses and MITS diagnoses are presented. There was concordant diagnosis and cause of death determined by MITS in 93.3% of stillbirths and 66.7% of neonatal deaths with an overall diagnostic concordance of 83.3%. However, when we used only histological features of MITS samples to evaluate the severity of the pathological features and assigned a cause of death, the overall concordance rates decreased to 62.5% (Table 4).

**Figure 1:**
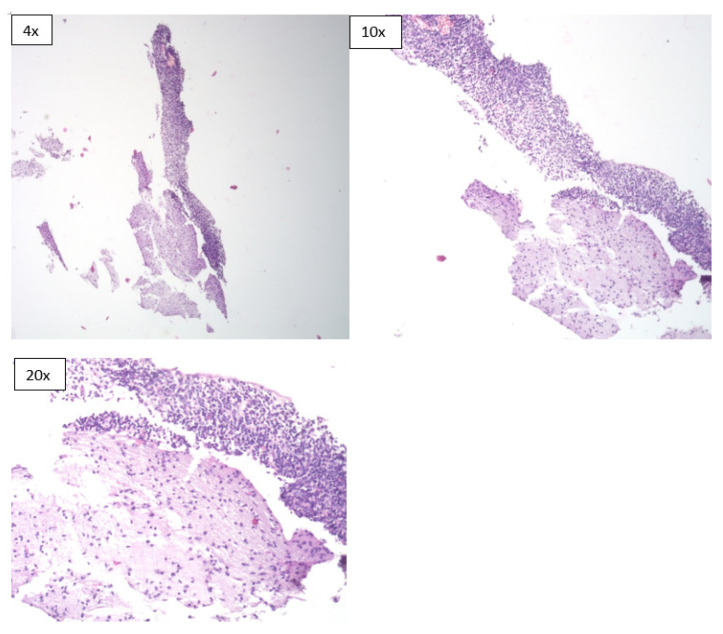
Microscopic sections of brain tissue obtained during the MITS procedure (H & E *). * Hematoxylin and eosin stain.

**Figure 2:**
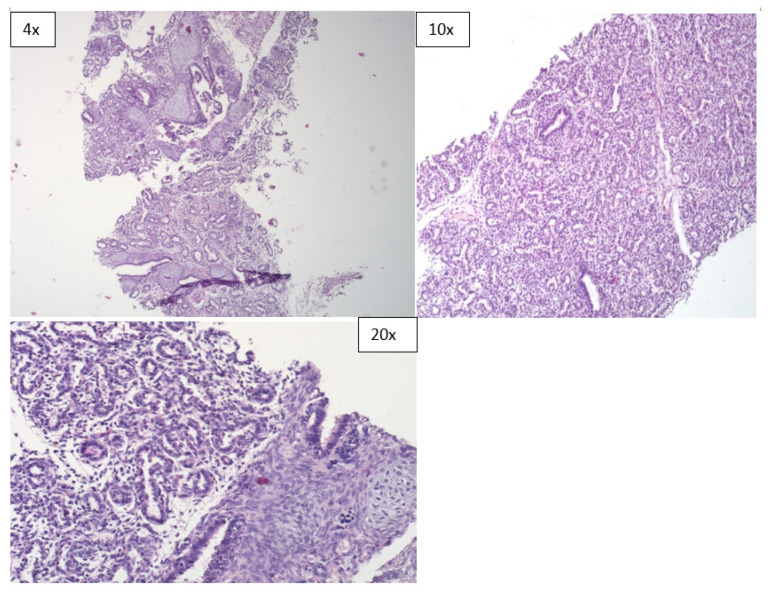
Microscopic sections of lung tissues obtained during the MITS procedure (H & E).

**Figure 3:**
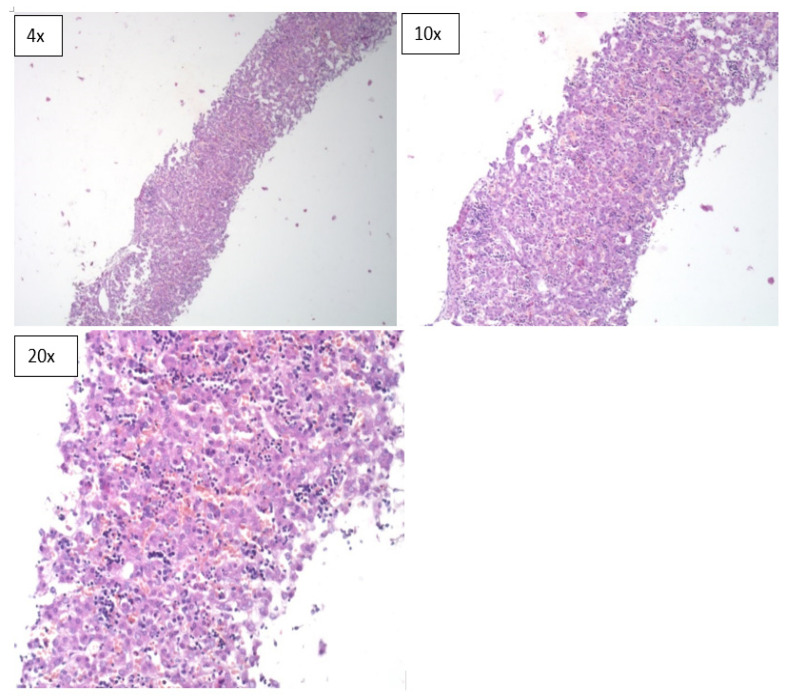
Microscopic sections of liver tissues obtained during the MITS procedure (H & E).

However, the diagnostic concordance of MITS based only on histological evaluation of tissue sections was limited. It was 22.2% in neonatal deaths, 86.7% in stillbirths and had an overall concordance of 62.5% (Table 4). In terms of specimen adequacy, the MITS method was able to provide adequate sample sizes for pathological evaluation from all the organs that were sampled (Figure 1, Figure 2 and Figure 3).

## 4. Discussion

This pilot study shows the concordance of MITS with CDA in identifying cause of death in stillbirths and neonatal deaths. Blinded evaluation of MITS samples alone was limited in providing sufficient information for assigning cause of death, especially in stillbirths and neonates with congenital malformations. When MITS samples were interpreted with antemortem clinical data including the results of laboratory tests, the immediate and underlying cause of death was attributed with confidence. Using this holistic approach to cause of death diagnosis, there was concordance with the CDA in 93.3% of stillbirths and 66.7% in neonatal deaths, with an overall concordance of 83.3%. We discovered that in both neonates and stillbirths, there was often multiple pathologies involved in the chain of pathological events leading to death.

Although a complete diagnostic autopsy remains the gold standard to determine cause of death (CoD), it is not routinely implemented due to various reasons: religious objections, sociocultural beliefs, limited resources and low demand from physicians and families. Nowadays, more than 5 million stillbirths and neonatal death occur annually, the majority of them in low- and middle-income countries, and for many of them, the cause of death (CoD) remains largely unknown [14]. In recent times, the world has faced emerging pandemics due to coronaviruses, whose pathology was not well-understood. Autopsy studies played a significant role in unraveling the pathology of these emerging pathogens. One recently published review paper written by Sessa et al. and Salerno et al. (2021) [2,3] presented a complete overview of autopsy in three coronavirus cases over the past two decades. This review involved 116 publications: 14 studies were collected concerning MERS-CoV, and 100 studies on SARS-CoV-2. In all cases, the autopsy provided much information about each unknown coronavirus. Authors concluded that, despite advanced technologies in all diagnostic fields, autopsy remains the gold standard method to understand the biological features and the pathogenesis of unknown infections, especially when awareness of a pathogen is restricted and the impact on the healthcare system is substantial. This clearly indicates that without autopsy studies, no significant information about the pathogenesis of these pathogens could have been known. Minimally invasive tissue sampling (MITS) is increasingly used in postmortem examinations for ascertaining the CoD in stillbirths and neonates [15]. Even though CDA is the gold standard in identifying the cause of death, one recently published multicenter study in Ethiopia confirmed that MITS could be a useful alternative method in the pathological evaluation and the determination of cause of death in preterm neonates [16]. Moreover, the MITS method (performed in Mozambique, South Africa, Kenya, Mali, and Bangladesh) was able to determine causes of death in stillborn fetuses and in deceased neonates and children younger than 5 years [17]. MITS also has the potential to address the knowledge gap on specific causes of neonatal mortality in situations where complete diagnostic autopsy is not routinely conducted in clinical practice. Additionally, owing to the MITS method, hospital-acquired multidrug-resistant bacterial infections were found to be the leading immediate cause of neonatal deaths in an observational study in India [18].

This is the first study in Kazakhstan that has been conducted to evaluate the validity of MITS in stillborn babies and neonates and compared the results with that of CDA. Although our cohort is small, our results show that there is a high concordance in cause of death diagnosis between these two pathologic approaches. There is a strong potential that when appropriately trained health officials in the technique conduct MITS, with adequate sampling of target organs for pathological, microbiological and molecular analysis, a cause of death diagnosis can be confidently determined in stillbirths and neonatal deaths. The mortality data obtained using MITS procedure seem adequate to guide the development and implementation of effective intervention strategies and improve current protocols for preventing intrauterine and neonatal death. MITS requires less time to perform, is less costly and more esthetically acceptable to pathologists and relatives of deceased children. In our study, we found that intrauterine hypoxia, congenital malformations and infectious diseases were the main causes of death in these premature babies and neonates. Overall, intrauterine hypoxia constituted 54.2%, with 100% diagnostic concordance between the two methods. Congenital malformations, deformities and chromosomal abnormalities were the second cause of death in our patients’ cohort, representing 20.8% of all the cases. In a previous study in stillbirths and neonates in Mozambique, intrauterine hypoxia ranked third in cause of death diagnosis, preceded only by fetal growth restrictions and infectious diseases [8]. A South African study found that congenital malformations were the leading cause of death (22.1%) in children 1–59 months of age [19].

We believe that the results of our study have some implications for strengthening preventive measures in our antenatal care services. With intrauterine hypoxia clearly being the measured cause of death in stillbirths, pre-conception counselling and measures to identify potential risk factors for pregnancy complications and adverse outcomes need to be improved. Screening for pre-existing maternal conditions and appropriate management of diseases diagnosed during the pregnancy course need to the improved. We also think that better pregnancy control such as careful and regular follow-up and an appropriate timing and mode of pregnancy termination needs further improvement.

Congenital malformations, deformities and chromosomal abnormalities are the next leading causes of death in our studies. In this case, we consider that pre-conception counselling, appropriate prenatal screening and diagnostic tests are important for better outcomes. The American College of Obstetrics and Gynecology Committee on Genetics has issued recommendations on preimplantation genetic testing for both screening and diagnostic purposes [20]. We believe that following these recommendations will improve pregnancy outcomes in this category. For infectious causes, we believe that timely diagnosis of specific etiologic agents and intervention according to guidelines, protocols and algorithms will improve pregnancy outcomes.

## 5. Conclusions

Perinatal and neonatal deaths are important indices that are used to measure the quality of the health care delivery services in any country. The burden of most of these deaths is usually higher in low- and middle-income countries (LMIC). With dwindling autopsy rates around the world, it is important to find alternative ways for obtaining vital mortality statistics to fill this gap. Mortality statistics are very crucial for global health development and for planning, prioritization and implementation of prevention strategies. MITS is accurate in the majority of cases (83.3%), less time consuming, requires less skills that would be required for CDA, and less expensive, thus saving funds that can be invested in other health care programs. We believe that CDA still remains the gold standard for cause of death determination and can be used on a case-by-case basis, but MITS can be employed in the majority of cases when CDA is either not possible or not consented by relatives, among other reasons.

We recognize some limitations of our study. The sample size is small and may not be adequate for validation purposes. Further analysis and more time for face-to-face gathering of information were hampered by the COVID-19 quarantine measures, suspension of or very limited access granted for research activities and the repurposing of the laboratory services for COVID-19 diagnoses. For this reason, we could not perform some molecular tests such as the PCR. Nevertheless, we successfully demonstrated the utility of MITS and its diagnostic performance when compared to CDA in pathological practice.

## Figures and Tables

**Table 1:** Baseline characteristics of the study deceased patients.

Variable	All, n = 24
Mother’s characteristics	
Mother’s age, mean ± SD	30 ± 5.9
Gravidity, n (%)	
None	4 (16.7)
One or more	20 (83.3)
Parity, n (%)	
None	7 (29.2)
One or more	17 (70.8)
Deceased patient’s characteristics	
Stillbirth, n (%)	
Yes	15 (62.5)
No	9 (37.5)
Sex, n (%)	
Female	8 (33.3)
Male	16 (66.7)
Weight (kg)	
mean ± SD	2244 ± 1420
median (IQR)	2201 (1250–3027)
Length (cm), mean ± SD	
mean ± SD	46.2 ± 11.9
median (IQR)	48.5 (38.3–53)
* Gestational weeks, mean ± SD	31.8 ± 5.5
* Age (days), median (IQR)	20 (13–34)
Time between death and autopsy (hours)	
mean ± SD	29.2 ± 25.3
median (IQR)	23.4 (10.7–39.5)

* Gestational weeks for stillbirths, while child’s age in days for neonates.

**Table 2:** Causes of deaths among all, stillbirths and neonates determined by complete diagnostic autopsy.

Cause of Death in Complete Diagnostic Autopsy, ICD-10 Code	All, n = 24	Stillbirths, n = 15	Neonates, n = 9
***Intrauterine hypoxia, n (%)***	13 (54.2)	13 (86.7)	0 (0)
P20.0 Intrauterine hypoxia first noted before onset of labor	11 (45.8)	11 (73.3)	0 (0)
P20.1 Intrauterine hypoxia first noted during labor and delivery	2 (8.3)	2 (13.3)	0 (0)
P28.0 Primary atelectasis of newborn n (%)	1 (4.2)	0 (0)	1 (11.1)
P36.8 Other bacterial sepsis of newborn, n (%)	3 (12.5)	0 (0)	3 (33.3)
E72.2 Disorders of urea cycle metabolism, n (%)	1 (4.2)	0 (0)	1 (11.1)
J16.8 Pneumonia due to other specified infectious organisms n (%)	1 (4.2)	0 (0)	1 (11.1)
***Congenital malformations, deformations and chromosomal abnormalities, n (%)***	5 (20.8)	2 (13.3)	3 (33.3)
Q89.7 Multiple congenital malformations, not elsewhere classified	4 (16.7)	2 (13.3)	2 (22.2)
Q91.0 Trisomy 18, meiotic nondisjunction	1 (4.2)	0 (0)	1 (11.1)

**Table 3:** Concordance rates of causes of deaths between complete diagnostic autopsy and MITS among all, stillbirths and neonates, employing underlining causes.

Cause of Death in Complete Diagnostic Autopsy, ICD-10 Code	MITS Concordance		
	All, n = 24	Stillbirths, n = 15	Neonates, n = 9
***Intrauterine hypoxia, % (Cohen’s kappa)***	100 (1.0) *	100 (1.0) *	-
P20.0 Intrauterine hypoxia first noted before onset of labor, %	100	100	-
P20.1 Intrauterine hypoxia first noted during labor and delivery, %	100	100	-
P28.0 Primary atelectasis of newborn	100		100
P36.8 Other bacterial sepsis of newborn, %	33.3	-	33.3
E72.2 Disorders of urea cycle metabolism, %	0	-	0
J16.8 Pneumonia due to other specified infectious organisms	100		100
***Congenital malformations, deformations and chromosomal abnormalities, % (Cohen’s kappa)***	80 (0.86) *	50 (0.63) *	100 (1.0) *
Q89.7 Multiple congenital malformations, not elsewhere classified, %	75	50	100
Q91.0 Trisomy 18, meiotic nondisjunction	100	-	100
*Overall, %*	83.3	93.3	66.7

* McNemar’s test statistic is non-significant and Cohen’s kappa test statistic is statistically significant.

**Table 4:** Concordance rates of causes of deaths between complete diagnostic autopsy and Minimally Invasive Tissue Sampling (MITS) based on histological investigations among all, stillbirths and neonates.

Cause of Death in Complete Diagnostic Autopsy, ICD-10 Code	MITS Concordance		
	All, n = 24	Stillbirths, n = 15	Neonates, n = 9
***Intrauterine hypoxia,% (Cohen’s kappa)***	100 (1.0) *	100 (1.0) *	-
P20.0 Intrauterine hypoxia first noted before onset of labor, %	100	100	-
P20.1 Intrauterine hypoxia first noted during labor and delivery, %	100	100	-
P28.0 Primary atelectasis of newborn	0	-	0
P36.8 Other bacterial sepsis of newborn, %	33.3	-	33.3
E72.2 Disorders of urea cycle metabolism, %	0	-	0
J16.8 Pneumonia due to other specified infectious organisms	100	-	100
***Congenital malformations, deformations and chromosomal abnormalities, % (Cohen’s kappa)***	0 (0) ^#^	0 (0) ^#^	0 (0) ^#^
Q89.7 Multiple congenital malformations, not elsewhere classified, %	0	0	0
Q91.0 Trisomy 18, meiotic nondisjunction	0	-	0
***Overall, %***	62.5	86.7	22.2

* McNemar’s test statistic is non-significant and Cohen’s kappa test statistic is statistically significant. ^#^ McNemar’s test statistic is statistically significant and Cohen’s kappa test statistic is non-significant.

## Data Availability

The raw data supporting the conclusions of this article will be made available by the authors, without undue reservation.
